# British Institutions for the Insane

**Published:** 1853-01-01

**Authors:** 


### THE JOURNAL

OF

### PSYCHOLOGICAL MEDICINE

AND

### MENTAL PATHOLOGY.

JANUARY 1, 1853.

### Art. I.—BRITISH INSTITUTIONS FOR THE INSANE.

In our last Number we gave a general outline of some of the principal lunatic asylums of North America, which contained many interesting facts and statements culled from the valuable reports drawn up by the medical officers of these establishments. On the present occasion, we propose adopting a somewhat similar proceeding, in regard to the numerous institutions for the treatment of lunatics, of which Great Britain may justly feel proud, as shown by the official documents now on our table, and with which we have been favoured by the physicians or superintendents of the different asylums.

Amongst so many instructive reports, it is difficult to select wherewith to begin our analysis; nevertheless, we think Yorkshire merits precedence, being the largest county in England, as also on account of the great importance of its lunatic institutions, whereby so much benefit is conferred upon the general population. The first we would notice is that of the asylum at Wakefield, and drawn up by Dr. Corsellis, already well known to the profession, who states that

"He has the satisfaction to record that the events of the last year afford a review of comparative prosperity. The inmates have been unusually free from serious physical disease. But one accident has occurred of sufficient importance to disturb for a short time the tranquillity of the household.* By far the larger portion of the inmates have appeared contented and happy; whilst all have been (so far as outward circumstances could render them) quiet and comfortable; and the curative results have not only equalled, but in some measure surpassed, former experience. Succeeding to the painful events of the

* A patient escaped while taking his usual walk, and drowned himself.

last quarter of the preceding year, and its protracted history of Asiatic cholera, such immunity demands from all concerned in the welfare of the institution the utmost gratitude to a merciful Providence, through whose beneficent care the accustomed peace and prosperity of the household have been restored.

"Discharges.—One hundred and twenty-one patients have been discharged, a few of whom were sent away for the trial of a month, and from whom satisfactory statements have been subsequently received of their continuance in health of body and mind; and not a few have borne witness to the salutary influence of the moral and religious feelings established during their residence in the asylum, by their own personal reformation of character, and the better discipline of themselves and families.

"The evils of a too early removal from the beneficial treatment of a public institution, although less felt in county asylums than in those to which the opulent classes have access, are nevertheless occasionally occurring. Friends are deceived by the apparent calmness and improved condition of the patient; the old and fallacious argument is urged, 'that if they can work in the asylum, they can do so at home;' they become importunate, and assist in augmenting the desire of the patient for a premature restoration to liberty.

"It is a fact known to all who have opportunity of observation, that many of the insane who are noisy and outrageous whilst under the care of their friends, become calm and docile when admitted into an asylum. Such a change does not indicate the cessation, or even (in some instances) the mitigation, of disease, but that it is held under control by the varied influences brought to bear on it. Many of our inmates who are peaceful and contented, cheerfully occupied throughout the day, entering with pleasure into the amusements and recreations afforded them, or rambling at will in the grounds of the asylum, would become unhappy and unmanageable, if restored to the exciting causes of their malady.

"In the treatment of other diseases it is not considered safe to suspend remedial measures on the first indications of their successful employment, or hastily to relax in that precautionary treatment of convalescence, which can alone result in complete restoration. How is it, that society reasons less correctly on insanity than on other diseases?

"The cerebral system is amenable to the same natural laws as other parts of the human frame; and if in other physical diseases the moral treatment forms a part, and no inconsiderable one, how much more important is the careful and persevering use of curative means in that class of maladies in which the organ of thought itself becomes the principal seat of disease.

"Instances not a few might be adduced from the journals, of relapses in which the patient has been brought back to the asylum sunk in despondency and self-renouncement, after having presented the most encouraging proofs of convalescence which might have been matured had sufficient time been allowed, before old associations and former exciting causes had been again encountered."

Dr. Corsellis subsequently adverts to the movement of patients in this asylum, upon which point he says :

"The admissions present an advance of 17 on those of the preceding twelve months: 285 patients have been admitted, of whom 49 have died. Some were brought in the last state of exhaustion, whose life and sufferings terminated in a few days; others, by the use of restorative means, lingered out a few weeks or months without any favourable symptoms.

"Amidst the disadvantages to which county asylums are especially subject is that of being the last instead of the first resort to the mentally afflicted. The hope of cure, without the imagined stigma of so declared and public an avowal as is involved in placing a relative in an institution for the insane, prevails to a greater extent even amongst the lower classes of society than might at first be believed; and every means within the reach of influence or funds is resorted to, mostly in vain, and often so injudiciously applied as to aggravate the evil it is intended to cure, until, funds and patience alike exhausted, the asylum becomes the last refuge to the sinking or incurable patient, and a relief to the anxiety and fatigues of neighbours or friends.

"In a former report it was stated that a cessation to the once numerous influx of suicidal cases had given a respite to the watchfulness formerly required. The two past years contain the record of no less than 133 patients admitted with suicidal propensity, suggesting the probability of epidemic influence in this phase of mental disorder. From the month of June last, 7 females have been received whose propensity to self-destruction has been particularly declared in a determined resistance of food. With a single exception, all were fed for a longer or shorter period by the œsophageal tube; the resistance has given way, and, with the above exception, they are progressing favourably."

Speaking of the general management of an asylum for insane patients, the reporter, in reference to the West Riding institution, remarks judiciously :

"So much depends on this division of the subject, that, notwithstanding it has been copiously treated of in former reports, and that nothing new remains to be set forth, it may yet be right to give prominence to the fact, that all the patients are employed or amused to the fullest extent compatible with their condition.

"Incomparably more is done by means of the confidence we are able to inspire through the moral influence we exercise over the 'mind diseased,' by obtaining the trust of those committed to our care, and their belief that both the power and will to benefit are ours, than by any specific course, either medical or moral. To establish and maintain confidence in the system, and confidence in those by whom it is carried out, to call forth a spirit of protective guardianship, of self-government and forbearance on the part of attendants, and of good will and thankfulness on that of patients, will do infinitely more in restoring their impaired powers, than all the rules that can be put together, or all the medicines of the pharmacopœia combined. From this cause it is that so much less inconvenience and difficulty is encountered with patients who have before recovered in the asylum. It is common to hear from such as have suffered a relapse, a desire to be with the 'old attendant;' a request always complied with, if possible; and in such as are subject to maniacal paroxysms at long intervals, those attacks are generally found to diminish in intensity, as well as to shorten in duration.

"In all places of reception for the insane, a like difficulty is felt in the way of furnishing employment for men; more especially in winter, when out-door occupations cannot be either regularly or extensively followed. With the females, this difficulty is unknown; and when their industry is observed in washing, sewing, knitting, cleaning, &c., the remark once made by the celebrated Robert Hall, that 'if he had known how to hem a pocket-handkerchief, he should never have been deprived of reason,' may be easily understood.

"A supply of cocoa-nut fibre, for picking, from the neighbouring house of correction, has furnished a useful occupation for the imbecile male patients. The unusual mildness of the season has also given great advantage, by allowing their labours in the grounds and gardens. Large numbers of men have been employed in trenching, levelling, and preparing for planting the ground in front of the auxiliary building, and in pulling down the walls dividing three airing courts attached to the east wing of the original one. A community always fluctuating, and constantly draughting off such as are set at liberty from the numbers of the useful and industrious, of course witnesses a continual change in the several workshops; there is, however, always a sufficient number employed to keep the looms in constant work, and to supply the institution with shoes and clothes, well made.

"As a reward for good conduct, as an encouragement to the depressed, and as a safe link of communication with the outer world, nothing has been more satisfactorily allowed than frequent walks into the country, of parties, sometimes men, at other times women, with the attendants. Nor have the parties for tea, music and singing, or dancing, been less numerously attended, or less pleasantly conducted."

The next public asylum to which we would direct attention is that of the North and East Riding, at Clifton, of which Mr. Samuel Hill is the medical superintendent. According to that gentleman's official report, the daily average number of resident patients was 236, of whom 150 were admitted during the year; the two sexes being exactly equal, or 75 of each. Of the admissions, the following summary exhibits the form of mental disease under which the new patients laboured—viz., 31 males and 45 females were the victims of chronic mania; 9 males and 10 females had recent mania; 11 males and 7 females were weak-minded; 8 males and 7 females were idiotic; 10 males and 3 females, epileptic, whilst 6 males and 3 females were afflicted with general palsy.

In addition to these particulars, it is interesting to mention, that 13 of the males and 11 of the females admitted were disposed to commit suicide; and lastly, that 8 male patients, with 6 females, died during the year, or 4·6 per cent. of those under care. Respecting the number of recoveries, Mr. Hill remarks :

"The proportion of cures from amongst so large a number of apparently confirmed cases of insanity, epilepsy, palsy, and idiocy, must be small; but change of habit in the dirty, the quelling of strife with the turbulent, humbling the proud, pacifying the daring and violent, reconciling the restless, exciting the drone to exertion, the apathetic to observation, the suicide to love of life, the homicide to dread of crime, the thief to an appreciation of honesty, the destructive to esteem value, the slothful to early rising, the melancholic to share in the enjoyments of the cheerful, the reserved to social communion, the mute to speak, the hypochondriac to obliviousness of the past, the dispirited and fretful to happiness, and the morose to civility, are attainments more or less to be achieved.

"To cure those who are placed under early treatment is often not very difficult : a large per centage of such patients ultimately recover, unless the insanity is complicated with some other disease; for example, out of 19 favourable cases admitted in 1851, 17 have been already discharged cured. On the 31st December, 1850, only 3 hopeful cases remained in the house, out of 154, so that not more than 22 of the number under treatment in 1851 were of the probably curable class—all of whom have now recovered."

In reference to the great object of curing, if not, of materially ameliorating, the afflicted condition of lunatics, when they are incurable, the report further says—

"As the aim of all treatment and management is intended to make the insane act, speak, look, and think as much as possible like persons of sound reason, it must be obvious that the system observed towards them should in all respects be simple, uncomplicated, and truthful, free from mystification, and be within the compass of their feeble and diversified powers of understanding. Some of them seem to be entirely mechanical in what they do—the will scarcely playing any part at first —but in time the mind is operated upon by employment, and the occupation becomes engrossing, a certain amount of thought and judgment being exercised. Such an amendment in an insane person inspires a hope, and sheds its influence over the destinies of others of the same lost class."

Amusement and recreation constitute likewise prominent objects constantly kept in view at this institution, as shown by the following paragraph :

"Change and variety are indispensable for the insane : cricket, music, dancing, evening parties—on which occasions from 160 to 180 of both sexes meet together in joyous harmony; fruit-gatherings in the summer, daily walks, and whatever can tend to animate, enliven, and cheer, are adopted as beneficial agents. The patients have been entertained, through the kindness of our worthy chaplain, by an exhibition of dissolving views; and many humorous and instructive subjects shown through the medium of an excellent magic lantern, generously lent by a gentleman in this vicinity. We have also to thank the Committee of the Blind School for allowing their pupils to contribute so much to the amusement and gratification of the patients on the occasions of their evening assemblies, both by instrumental and vocal music."

The next establishment which comes under notice is the far-famed Retreat, near York, of which the 56th report now lies on our table. According to this document, the average number of patients resident during the past year was 113; the admissions having been 6 men and 6 women; whilst 3 males and 2 females died. A striking feature which is mentioned respecting the admissions deserves notice—namely, that 8 out of 12 new cases received were affected either with acute mania, or in a high state of excitement approaching to that form of mental disease. It is also worthy of record, that all have been managed without the application of any mechanical restraint; and although for several years previously acute mania had constituted a small proportion of the various forms of insanity then admitted, during the last 18 months the number of cases of that kind had exceeded the average.

The unusual attraction of the great exhibition seems even to have penetrated within the quiet precincts of the Friends' Retreat, seeing—

"Two small parties of male patients visited the Exhibition, under the care of competent attendants, and it may be readily inferred, derived great pleasure from the trip.

"Several parties also took an excursion to Scarborough in the course of the summer.

"In the winter, the lectures and experiments in natural philosophy proved as attractive as usual, and I believe the household would relinquish with great reluctance this pleasing and instructive variety in the way of spending some of the long winter evenings.

"The regular occupation of the patients has been also steadily attended to. Besides the ordinary kinds of work usually carried on, the preparation of the ground, and the wheeling away of materials for the commencement of the additional buildings, have furnished new and suitable occupation."

These remarks are satisfactory; and the managers of this institution may justly consider the retrospect of the past year affords them the consoling reflection, that their laudable efforts have not been relaxed to conduct this establishment in accordance with right principles, and to carry them out with steady perseverance.

Like Yorkshire, there are several public institutions for the insane in Lancashire, one being the County Asylum, of which Dr. de Vitré is the visiting physician. In the annual report of that establishment, dated July, 1851, it is stated, that

"During the twelve months ending on the 23rd of June last, 88 patients have been admitted; a diminution, as compared with the preceding year, to the amount of 125; though the daily average number, being 773, is higher than at any period since the opening of the establishment.

"It will be observed that, the recoveries are less in number than usual this year, a circumstance almost entirely owing to the falling off in the admissions, from which, of course, a majority of cures are to be expected. It is gratifying, however, to be able to state that the deaths have been reduced to 8 per cent.—a rate of mortality below the average, both of this and most similar institutions of the kingdom. Several of the deaths have occurred amongst the older residents in the asylum; in three instances, the period of residence had been upwards of thirty years, and in four cases it varied from twenty to twenty-six years.

"On the opening of the two asylums at Prestwich and Rainhill in January last, relief was at once afforded to the various Unions in the county, by the admission of urgent cases, which had, of necessity, been detained in workhouses, at great inconvenience, owing to the crowded state of both Lancaster and Haydock-Lodge Asylums."

Respecting the treatment adopted, Dr. de Vitré subsequently observes:

"There has been during the year no variation from the principles laid down in former reports. As the majority of the men have been accustomed to manual labour, it is usual to find them joining with alacrity in out-door occupation, either for the whole or a part of the day; and for those who are physically disabled, or cannot be trusted with implements of husbandry, daily exercise is afforded in the grounds surrounding the building, where they walk, under the care of attendants, in parties of about thirty. This arrangement is plainly much to be preferred to the use of airing-courts, as it must be obvious to the most casual observer that exercise within narrow limits soon becomes tedious; and, without great vigilance on the part of the attendants, too many may be seen lounging in unsightly attitudes, or escaping from observation when practicable."

During the year embraced in the official document now under consideration, it appears that the total mortality amounted to 62 patients, 32 being males and 30 females. Several interesting particulars in reference to the causes of death are given in one of the tables attached; amongst which, the most important features appear to be, that 11 women died of disease of the brain or its membranes, and not one male patient; whilst general paralysis proved fatal to 9 men, whereas, amongst the females none died by that malady. Phthisis was the cause of death in 5 men, against 10 females who died by that disease; and lastly, 2 men were the victims of epilepsy, but no female. The morbid appearances ascertained in 33 cases of insanity examined after death, are minutely detailed in a table subsequently appended, which will amply repay perusal, since it contains highly instructive information respecting the pathology of mental diseases.

The new County Asylum at Rainhill having been only instituted on the 1st of January, 1851, the official document now on our table is, consequently, the first annual report of that establishment. From this publication it appears, that 393 patients were admitted, 47 discharged, 1 escaped, and 48 have died; hence showing a per centage of 11·9 of cures, and 12·21 of deaths. With reference to the assigned cause of insanity in the 48 cases of death reported, intemperance seems to have been the most frequent; 16 examples of that description being recorded, whilst 7 arose through poverty, and 3 from domestic unhappiness. Again, with regard to the apparent causes of death in the fatal cases, it becomes interesting to ascertain by the report, that

"With the exception of severe diarrhœa, which made its appearance in August, and proved fatal to several of the inmates, we have not been visited with any sickness of an epidemic or contagious character. Ten of the deaths occurred within one week after the patients' admission into the asylum, and the greater part within three months. No less than nineteen were cases of general paralysis or supervening apoplexy; seven died from pulmonary consumption; five from the gradual decay of the vital powers; eight from maniacal exhaustion; and the others from the various causes above specified."

Similar to many county asylums, patients afflicted with epilepsy seem to have been very numerous at this institution; indeed,

"The number of epileptic cases under treatment during the year has been unusually large, and, from the severe nature of the attacks in several instances, they have been a source of great anxiety to the medical officers, as well as to those more immediately in attendance upon them. Being desirous to try all possible alleviatives in such cases, the extract of *cotyledon umbilicus* was used for a time, with much apparent benefit; but in every instance, after a variable period of quiescence, the attacks reappeared with increased violence. The tincture of sumbul is now being tried with a like motive; but the short period of time that it has been in use will not yet warrant any opinion on its merits."

Whatever result may follow the use of these medicines in so intractable a disease as epilepsy, we trust Mr. Eccleston will inform the profession in subsequent reports; and as that practitioner appears zealous in his endeavours to alleviate this incurable complaint, we would recommend him to try, amongst other remedies, the "valerianate of zinc," which has been recently mentioned by Dr. Webster, in his Notes on French Asylums, to have been found beneficial by some psychological physicians of that country.

Notwithstanding the acknowledged improvement, which even the general public admit has resulted to lunatics by the disuse of mechanical restraint, it is lamentable to hear that, prior to admission into asylums, many unfortunate victims of mental disease are still tortured in this manner. Thus, after alluding to a poor girl, who for upwards of two years had been kept in constant restraint by a strait-waistcoat, in consequence of being considered a dangerous lunatic, the report states that

"Another case, admitted in September, 1851, was securely bound by an ordinary cart-rope, the removal of which gave no little trouble from the complexity of its attachments, a strait-waistcoat and a pair of transport leg-chains completing the arrangement for the poor fellow's torture. He also was described as a most dangerous lunatic; yet in the afternoon of the day of his arrival he was working on the farm with his fellow-patients, and has not been absent from employment a single day since that period."

Instances like the above are very shocking; and indicate how much yet remains to be accomplished towards ameliorating the often unfortunate condition of persons attacked by insanity.

Although the Manchester Royal Lunatic Hospital is at present devoted exclusively to the care and treatment of the insane, belonging to the middle and higher classes of society: still, seeing it publishes official reports the same as county asylums, we therefore consider all such establishments to come legitimately within the scope of our present analysis of British institutions for lunatic patients. According to the second annual report, now on our table, the number of new cases admitted during the past year was 33, whilst 21 were discharged, of whom 17 left the hospital cured: thus giving a ratio of 51·22 per cent. upon the number of admissions. Besides the above results, 8 died; which makes the mortality 24·24 per cent. according to the same mode of calculation. Being a new establishment, the number of inmates has hitherto remained small; the average during the year being only 36, whilst the total patients under treatment amounted to 66, since the date of the last annual report.

Respecting the system pursued at this hospital towards ameliorating the condition of its insane residents, we would refer to the remarks of Mr. Dickson, the medical superintendent, who states, that

"All the means of cure, medical and moral, hitherto in operation, have been continued with increased vigour during the past year; and, notwithstanding the difficulties already alluded to in the beginning of this report, it is in the highest degree satisfactory, that I am enabled to report the great success which has attended my exertions.

"In all other physical diseases, the moral treatment forms a not unimportant part of the treatment, but it becomes of much more importance as one of the most essential elements in treating that class of maladies where the organ of thought itself has become the seat of disease.

"Of the means in use, active occupation in the open air is undoubtedly the most beneficial as a means of cure. The object kept in view in devising occupation for the patients, and in which they are engaged only and in so far as it is found to be remedial and a means of recovery, and which to a certain extent I have succeeded in impressing them with, has been to induce them to take that interest in the improvement of the gardens and pleasure-grounds, and in the progress of our farming operations, as would give a zest and object to their labour. This feeling has been developed, and displayed in the interest manifested by them in their various employments, and by the cheerful assistance given by them in the repairs and alterations necessary in the hospital.

"In addition to the ordinary cropping and gardening occupations, undertakings in the way of forming new walks, levelling, planting new fences, and other works, have been in constant operation, and have afforded occupation to the able-bodied, from the more intelligent and docile to the most imbecile and unmanageable. These operations have been principally carried on, on the piece of land immediately behind the hospital, which we are now laying out as a kitchen-garden, the site of which as originally planned having been found objectionable in certain respects. In addition to this, I have about six acres of land under crop.

"During the summer months, nearly two-thirds of the male patients have been regularly employed in out-door occupation during stated hours of the day (viz. four hours), under the constant care, inspection, and instruction of well-qualified attendants; in our hay and harvest operations the patients have given us much and valuable assistance, and during the summer even the ladies have frequently volunteered their aid, and have afforded us bands of cheerful and active assistants in our pleasure-gardens, although for them the chief source of employment is in the work-room, where, under the instruction and direction of the matron, the number of articles made and repaired sufficiently attests the activity and industry which prevails in the ladies' department. In addition to the more ordinary, they are also engaged in the more ornamental kinds of needle and fancy work. The habits of self-control, order, and propriety, are by these means so encouraged and restored, that they supply us with an important instrument for establishing that influence and control over the patients, so necessary and so conducive to their recovery.

"Music, singing, and drawing, are much practised; excursions are made frequently to some of the neighbouring towns and villages, for the purposes of shopping, &c., and occasionally to Manchester by a select few, to some of the popular amusements. These excursions are sources of great interest and pleasure. They form, from time to time, new subjects of conversation, and afford, with the means of in-door amusement, such as bagatelle, chess, &c., and reading from the library, a great and pleasing variety.

"The occupations of the gentlemen are more varied and numerous; in addition to the out- and in-door means of recreation already mentioned, they have access to the carpenter's and engineer's workshops.

One of our patients constructed a turning-lathe, every piece of which was, with remarkable ingenuity, manufactured by himself from the rough materials found upon the premises. He put himself systematically to work, made all his own tools, converted the cast-iron bars into steel, made moulds and castings of the different grooves, screws, and wheels, cut the frame and blocks out of the solid oak, and succeeded in constructing a lathe, upon which has been done all the turning work required on the premises."

The Staffordshire General Lunatic Asylum is the next institution to which we would direct the reader's attention. At this establishment 179 new cases were admitted during 1851, including both private and pauper patients, whilst 46 were discharged recovered, or 25·70 per cent., and 38 died, or 21·22 per hundred admissions, besides which 1 escaped; the number of lunatics remaining in the asylum being 349, on the 31st of last December.

To illustrate the views entertained by the authorities of this well-managed public asylum, we copy the following quotation from the report of Mr. Wilkes, the able resident medical superintendent:

"The universal testimony of all connected with institutions for the insane, both in England and elsewhere, shows that everything depends upon early treatment; that the recoveries in cases removed to a proper asylum within one month after the symptoms of the disorder have shown themselves, are upwards of 80 per cent., and even within 3 months are between 70 and 80, but that they diminish in a fearful proportion as delay takes place, and that when the insanity has existed from 6 to 12 months before admission, the recoveries are found to be diminished more than one half.

"The unfavourable circumstances under which the insane poor are placed in their own homes, are hardly mitigated when they are removed to the workhouse, and the number of cases brought to this asylum in handcuffs, legstraps, and various descriptions of restraint, bearing upon their persons marks of the rude treatment they have been subjected to, were alone, on the score of humanity, reason sufficient for avoiding this course; but on the score of economy the arguments in favour of the early removal of insane persons to an asylum are equally strong, though at first sight the reverse may appear to be the case. The weekly average cost in the workhouse is known to be small compared with the asylum; the case may possibly be a recent one, and unattended with disposition to violence, and a trial in the workhouse is decided upon. No change for the better takes place; the disorder gradually developes itself; some act of violence is either committed or dreaded; and the patient is fastened hand and foot, tied down in bed, in a position scarcely admitting of motion, and left, perhaps, to the mercies of two or three able-bodied paupers.

"Many of these cases sink under the disease or the treatment, and either die in the workhouse or are removed to the asylum almost in a dying state. Others, perhaps, live on; the disease becomes confirmed, and they are kept in the workhouse until their offensive habits, sinking health, or violent conduct, cause them to be removed to the asylum, where they remain a burden to their parish for life.

"The practice which frequently prevails in this county of sending recent cases of insanity in the first instance to the workhouse, is attended with great evil, and on no ground does it appear to be necessary. Besides causing delay, and often producing irritation and excitement in the patient, the treatment too frequently resorted to seriously aggravates the malady, all which would be avoided by the direct removal of the patient to the asylum."

Respecting the Northampton General Lunatic Asylum, of which Dr. Nesbitt is the superintendent, we cannot do better than transcribe from the last annual report the observations of that physician, on the medical statistical tables for the past year, which exhibit,

"That at the close of 1850 we had on the books of the establishment 259 patients, that we have admitted 106, consequently that the medical treatment has embraced 365, whilst on the 31st of December last, there remained in the house 268. The results of this treatment show, that 29 have been discharged recovered, and 10 have been relieved, that 25 left us unimproved, and as such were remitted to the care of their friends, or transferred to other asylums, that 1 escaped, and that 32 have died. The daily average number of patients having been 265, the mortality is thus 12 per cent.; of the number so dying 22 were males, and 10 were females; the average age attained by the females being something over 58, whilst that of the males was something under 48; this disadvantageous contrast being referable to the much greater proneness to epilepsy and general paralysis of males over females. The specific causes of death, with the age and bodily condition on admission of the patient, being set forth in the mortality table, do not call for any remark here. It may be permitted to observe, however, that the general health of the inmates has not been subjected to any unusually disturbing causes, and that we have been in mercy spared the visitation of all epidemic disease.

"Of the 106 admissions, whilst 66 have been union, no less than 40 have been private patients, of whom 24 were gentlemen and ladies, in the usual signification of those terms. The union patients have been, with one or two exceptions, all received from parishes in the county of Northampton, and full as the house has at all times been, it is worthy of note, that in no case have we refused admission where the proper credentials were produced to show that the party belonged to the soil of the county.

"The social condition of those admitted shows that 53, or one half, had contracted matrimony; and a further analysis shows, that whilst the average age of the single was 33 years and four months, the average age of those who had married was within a fraction of 45. The same relative portion holds good of those who died, the married having attained a mean average of 55, whilst the single just extended over 46; thus showing apparently the truth of what has been demonstrated by the insurance offices, that life, whether in or out of an asylum, is extended by the ratification of the connubial tie.

"I have not attempted to set forth in any tabular form, the phenomena that throw light on the prevalence of insanity—the causes that in a highly civilized state of society are rife in developing the disease, and those exhibiting its dependence on hereditary transmission. Nothing is more involved in obscurity than is the tracing of the true cause; the assigned one being too often the rude offspring of conjection. An enumeration of all the known physical and moral, would still leave a large hiatus to be filled up by the unknown causes, and the account would therefore lose much of its interest, whilst the deductions would often be at the sacrifice of truth. The same applies to hereditary transmission, and will continue to do so, as long as there is associated in the human mind the sentiment that insanity is the most serious reproach that can be fixed on a family."

From the rather meagre report of the medical officers attached to the Bedford Lunatic Asylum, we collect the following facts regarding the movement of patients in that institution during the year 1851. Thus, 78 lunatics—37 males and 41 females—were admitted: 26 were discharged recovered, 23 left improved, and 4 not improved; whilst 16 males and 11 females, or a total of 27 inmates, died; of which the causes seem to have been mostly general decay and epilepsy. The mortality was, however, less during the last year, which the medical authorities attribute to the dry summer and autumn. They also state that the general health of the patients had been remarkably good, and, farther, no serious accident had occurred. At the date of the report—viz., Feb. 2, 1852—there remained 267 patients in the asylum, or 131 males and 136 females, the majority of whom were deemed incurable. Another feature at this asylum also deserves notice—namely, 32 were epileptic patients, and 50 idiots, many being of dirty habits. In concluding this brief allusion to the Bedford Asylum, we must express our dissent to the paragraph in which the reporters say—"Several patients have been subjected to mild restraint, and we are still of opinion that, in some cases, such restraint is the best and kindest mode of treatment." Seeing that the names of Mr. Harris and Mr. Matthews are appended to the document containing such opinions, we of course presume they are not only sincerely entertained, but the result of their experience.

Unlike the document now passed under review, the report of Dr. Kirkman, physician to the Suffolk Lunatic Asylum, enters more into details, and therefore will repay perusal. During the year 1851, 91 patients were admitted into this establishment, 36 being males and 55 females, whilst 51 were discharged cured, being upwards of 56 per cent.; and 35 died, which gives a mortality of 38·46 per cent., if also calculated according to the admissions. Again, the report states:

"The house has been throughout the year very healthy, and, till quite the close of it, the mortality very sensibly decreased. Within the last few months there have been six admitted either in an exhausted state, or in very old age, and three so completely sinking, that no other statement of their existing condition could be reported than 'in articulo mortis.' It is a great question whether patients beyond the age of 70 should be removed from their homes to asylums at all; and when the evident phase of disease is merely senile imbecility, and that of the most harmless character, only requiring the care of an attentive nurse, it is unjust to the institution by increasing its mortality to send them; and the removal, often from a considerable distance, injurious to the patient himself. An old man was admitted on November 28, aged 82, who could only be supported, or indeed kept alive, by beef-tea and wine: he died in a fortnight. We had another of 72, another of 70, requiring just the same treatment, but ending in the same result.

"The number of patients who have been almost uninterruptedly employed throughout the whole year has been more than usual; and they have done more work generally, and made more particular improvements than we have ever been able to effect before. The airing-courts have been completed which were begun last year; the walls throughout the whole range of external buildings have been lowered from the top, and the earth has been excavated on the inside; so that, whilst as regards their actual height the chance of escape is not greater than before, *now* from almost every court an extensive view of the surrounding country is obtained and enjoyed by those who have themselves laboured to be relieved from the sight of brick walls alone. With the bricks thus obtained they have built a wall of four feet high completely round the house garden, and an inspection room for an attendant in one of the courts; and have enlarged and improved the piggery and the farm. This work they engage in so readily, that it would hardly be unreasonable to view it as labour not altogether lost, to pull down a wall, only for the sake of building it up again. In such an establishment as ours, however, without resorting to this expedient, there are always external alterations or improvements to be made, not only justifiable on the score of employment, but sufficiently *necessary* for the convenience of the house.

"Many of our apparently most unpromising patients have become convalescent; many of our convalescents have got perfectly well, by this self-selected occupation; and many more, whose affliction had assumed a chronic character, have experienced that the asylum, as a house of industry, has comforts even for them. The mind, like the body, spontaneously impelled to exertion by the example of all around it, not only involuntarily forgets its pain, or its sorrow, but ceases to be what it was before—a power degraded to habitual inertia, for want of external excitement. Thus, by pleasurable, and therefore profitable engagement, ordinary cases rapidly advance to their cure; deliverance is constantly obtained from the otherwise uncontrollable paroxysms of variable mania, and relief diminishes that weight of darkness which is the characteristic of a gloomy melancholy."

In a subsequent page Dr. Kirkman judiciously remarks:

"It is indisputable that proper employment must always form a prominent part in the salutary treatment of the insane; it is the curative process in *many*, the consolatory process in *most*, and an advantageous process in *all*. The employed are amongst the well-disposed and quiet, the unemployed amongst the disturbing and disturbed. It has indeed been alleged by some who idolize optimism, rather than study practicability, that there should be *no* patients unemployed. This is undeniably the only limit to which our exertions tend; but still it must *necessarily* be a valuable desideratum, rather than a feasible result, both on account of cases which *cannot* be employed, as some *idiots*, and of others which *will* not, as those whose more evident manifestations chiefly consist in obstinacy and idleness. These are, however, often overcome by persuasion, as is the case with one of the best assistant bricklayers, perhaps, about this neighbourhood—a man who, with scarcely any exception, was the most intractable, suspicious, and dangerous, of the male patients of the house. He is now always quietly at work in the different engagements that arise. We now contract for paint, and the patients paint the house—for glass, and the patients glaze it; and in their varied employments, shoe-making, &c., their work is as cleverly done as it could be by sane agents: a fact which, we might observe in passing, illustrates the truth that mental aberration seizes most upon the abstract functions of the mind—upon the moral ideas rather than upon adventitious knowledge, and from which we might draw several interesting deductions."

On the subject of religious instruction, the same authority says:

"The patients attend divine service in the chapel, as usual, which, as reported before, is too small for the numbers: perhaps in another year our labourers may be able to accomplish some improvement here themselves. The subject of religious instruction is too delicate to be discussed in an ordinary report. It will be enough to convey the repeated conviction, that to be really effective, *professional* can never supersede *domestic* instruction; the administration should be in that guarded manner which is only learned by the knowledge of the existing peculiarities of the parties addressed. Moral delinquencies may arise from mental idiosyncrasies, which need great forbearance and a peculiarly delicate mode of conveying 'instruction in righteousness.' A patient went home well, whose relapse after a former discharge 'came on,' as he expressed it, 'after hearing an alarming sermon:' his morbid conscientiousness was morbidly acted on, and he left the church to cut his throat. He is now well again."

With these quotations we take leave of the Suffolk Lunatic Asylum and its experienced physician.

For the county of Kent a large lunatic asylum has been erected, into which the annual medical report for the year ending July 4th, 1851, informs us, that

"126 male and 160 female patients, together 286, have been admitted, which, added to 396 remaining at the end of the previous year, make a total of 682 under treatment in the whole period; 48 of the patients admitted, being about one-sixth part, were suffering from repeated attacks. Again, 61 men and 73 women, together 134, were discharged or died, leaving 238 men and 310 women, together 548, on the 4th of July; whilst of the above, 22 men and 27 women, 49 in all, were discharged recovered; one of the men and three of the women having been absent from the asylum, on trial, during convalescence, previously to their absolute discharge; and lastly, 35 men and 30 women, together 65, died."

Respecting the all-important question of personal coercion, the medical officers, Drs. Sibbald and Huxley, say:

"Restraint, by instruments worn on the person, has again been used to a small extent; the experience of the year now under review having upheld the views and opinions respecting the advantages to be derived occasionally from its mild application, upon which we had acted in former years. We have reason to be convinced of the therapeutic value and humanity of this agent, in a certain small class of cases, when its use may be entirely divested of harshness or the risk of inflicting bodily injury, and of needless duration or repetition; we are inclined to place it in the list of indispensable adjuvants to treatment, whilst we believe it to exert sometimes a direct remedial agency of its own of considerable value.

"As we are led to imagine that there is a want of accurate information with regard to the use or disuse of restraint in the county asylum, and to think that, in the absence of any public explanation, there may be a general idea that restraint is a thing of course in such an institution, we desire, in a few words, to place the truth of the matter before the magistrates. The readiest means of accomplishing this will be, an exact return of all the instances wherein restraint was resorted to in the practice of the asylum during the last year, premising that, except for strictly medical purposes, restraint has not, for several years, been used in this asylum at all. There is a wide difference between the use of restraint remedially, and the general employment of it merely to control violence. The latter alone can, and must, unavoidably, give an unhappy character to the whole economy of an asylum. Besides the direct physical injury of it to the patients, there could be no worse effect than the tone, temper, and feeling which the habitual resort to it would create in the minds of the attendants for their patients, sufficient for ever to prevent the only true advancement. Hence the necessity for a distinction entirely founded on the principles guiding the choice of the instruments of restraint, and especially distinguishing the objects and manner of their application.

"It is scarcely necessary, at the present time, to abjure all restraint, if any true uses it may possess have been overlooked in the course of its general abuse; although the public are much indebted to the advocates of entire non-restraint for the speedy change wrought by them in a system at once mistaken and cruel, and so universal as to require for its destruction the weight of uncompromising public opinion."

Besides the subject of mechanical restraint, the above authorities likewise advert to the treatment of lunatics by seclusion; and upon this point they inform us that

"Twenty-nine men and sixty-two women were placed in seclusion for shorter or longer periods, on various occasions during the year. The total number of hours so passed by the men is 1886, or sixty-five hours altogether, by each man. The total number of hours of seclusion for the women is 2979, or forty-eight hours for each woman. Periods of seclusion vary from an hour to a day, and are always terminated as soon as their object may be secured, which is the restoration of the secluded person to the ordinary tranquillity of mind, permitting association with the other patients in a ward without danger or too great disturbance."

These facts are significant; but we wish it had been likewise added, whether such proceedings really proved beneficial or otherwise, in order to serve as a guide for other practitioners.

As the annual report of the Gloucestershire General Lunatic Asylum contains no remarks whatever of the medical officers, although some useful tables are appended, we therefore extract from the official statement published by the visitors the subjoined observations, which are interesting:

"The course of treatment pursued towards the patients, both mentally and bodily, has tended much to their comfort and general amelioration; and has been so far successful, as to cure, that the tables show that while the admissions during the year have been 128, the number discharged as cured have been 66. At the close of the last year there only remained in the house, out of 300 patients, 31 who were deemed to be curable, and there are now 26; so that the cures may be considered as exceeding 50 per cent. on the admissions of the year. The general health of the patients has been most satisfactory, and there has been an entire absence of any epidemic during the year: the mortality, however, has been unusually great for this institution, amounting to 45, a number considerably exceeding that of last year, and very much larger than that of former years. The visitors have no means of accounting for this increase, except that, owing to the extraordinarily small mortality of the two or three years preceding 1850, a number of shattered constitutions had out-lived the previous years to swell the mortality of 1850 and 1851, and also the circumstances referred to specially in their last report—namely, the very aged and feeble state in which many cases are received into the asylum. During the past year five patients died within three weeks of admission, one of whom was upwards of eighty years of age, two more upwards of seventy, and others above sixty. The visitors think it necessary to refer more particularly to one death, which occurred in the institution in September last, under the following melancholy circumstances. A gentleman who had been an inmate of the asylum many years, usually of quiet habits, but subject to occasional fits of excitement, who had retired to rest tranquilly and in his ordinary health, appeared to have risen from his bed in the night and made an attack upon his window, which he succeeded in breaking through, and precipitating himself to the ground, a distance of more than forty feet, by which his death was caused. The patient had never shown any suicidal propensity, and it would seem very doubtful whether he had any idea of the kind in this instance, or was conscious of the act he was committing. These window frames, attached to the bed-rooms of the opulent patients, had been in use since the opening of the asylum, nearly thirty years ago, and nothing had occurred before to make them appear insecure. An immediate order was made for removing the wooden frames and replacing them by wrought iron of the same pattern. The visitors have the satisfaction of stating, in support of the system of the absolute abolition of every species of mechanical restraint, which has been adopted now for many years in this asylum, that before this unhappy occurrence only one case of suicide had occurred here for upwards of five years and a half, and that the unhappy event above mentioned can in no way be connected with such a cause, as under no circumstances would this unfortunate gentleman have been considered a subject for restraint.

"The general comfort and cheerfulness of the patients have been much promoted during the past year by frequent entertainments afforded to the paupers, as well as other inmates of the house, by Dr. and Mrs. Williams, who have spared no exertions or trouble in making these recreations useful as well as agreeable. The visitors think they ought to express in this report how highly creditable they consider it is to the superintendent that the discipline, cleanliness, and general health and comfort of the establishment, have been maintained in a most satisfactory state during the past year, in spite of many untoward circumstances, which have much increased the ordinary difficulties of management, such as the over-crowded state of the wards, many of which have had nearly double their proper allowance of inmates, who have been accomodated there temporarily, in anticipation of the speedy opening of the new female pauper wing; and secondly, the lamentable want of water under which the institution laboured for fifteen weeks, when, the supply from the Water Company and the wells having failed, they were dependent upon such an allowance of water as could be obtained by hauling from a brook distant half a mile. This evil has been of so very serious a nature, that had not the officers of the Water Company promised to procure from the Severn a full supply by another year, the visitors would have felt compelled to have pressed upon the magistrates of the county the necessity of procuring a sufficient independent supply for the asylum, although such could only be secured, it is believed, at a very great cost."

The Wilts County Asylum, of which Dr. Thurnam, so favourably known to our readers and the profession, is the medical superintendent, having only been opened on the 19th of September, 1851, for the reception of patients, the report now before us hence embraces a limited period. Nevertheless, it is satisfactory to learn—

"The superintendent must briefly express the gratification he experiences in reporting the material improvement, in the bodily condition as well as in the personal habits and conduct, of a large proportion of the patients admitted into the asylum. The reaction of this amendment on the mental condition has been most obvious, particularly in the case of many of the female patients. Habits of order, regularity, and propriety have to a great extent been established even in the most confirmed idiotic and demented patients, and further improvement in all these respects may be confidently anticipated.

"The employment of the patients is an object of primary consideration. A large proportion of the men are engaged in agricultural pursuits, under the care of an out-door attendant. It is intended still further to encourage this healthy and in every way beneficial occupation, as most in accordance with the previous habits of the majority of the patients. Some efforts have also been made to employ the male patients in other ways. A tailor and a shoemaker-attendant have both been engaged; and it is hoped very shortly to have the workshops for tailors, shoemakers, and carpenters, brought into use. Under the active and judicious superintendence of the matron, an increasing majority of the female patients are occupied in the domestic labours of the kitchen, laundry, and wards, and in needlework. The greater part of the bed and house linen, and much of the clothing, has been, and is in process of being made by the patients, with the assistance of a single seamstress."

Subsequently, Dr. Thurnam observes, in reference to a question highly interesting to all Christians, that

"The chapel was opened for Divine service on the Sunday after the first admission of patients; and since that time they have regularly attended daily morning prayers and two services on the Sunday, in the afternoon of which day a sermon is preached by the chaplain. The proportion of patients attending chapel has generally amounted to 80 per cent., or four-fifths of the whole number. The effect of this regular attendance on Divine worship appears to be highly beneficial. A reference must not be omitted to the kind interest which the chaplain has evinced in the welfare of the patients, and the attention which he has paid to such of them as the superintendent has recommended to his especial notice."

At the Somerset County Asylum, of which Dr. Boyd is superintendent, 122 patients were admitted during 1851; 61 were discharged, and 40 died; thus giving a mortality of 32·78 per cent. in reference to the admissions; the total population at the end of the year being 340 lunatics. From the fourth report of this institution, recently published, we perceive that

"The most remarkable feature in the admissions during the year has been the number of suicidal cases; 12 males and 24 females were reported to have suicidal propensities, and some were brought in after having made most determined and deliberate attempts upon their lives; 3 males were suffering from wounds in the throat, one of whom is unable to swallow, and has to be fed by means of a tube; 7 females were reported to have made similar attempts; 6 had attempted drowning; 5 hanging; 2 strangulation; 2 leaping out of windows; and 1 poisoning. Some of these cases still require the strictest watching and attendance, and with many it has been found necessary to force their taking food."

Again, the same authority says—

"The classification of patients is most essential to their comfort and to the quietude of the asylum, particularly as regards the females, whose occupations in doors (unlike the males) bring them unavoidably more together. With this view it would be very desirable to make a sub-division amongst the curable and industrious patients, by separating those who are talkative and otherwise annoying from the quiet and convalescent, without placing these with the idle or mischievous class. The females are at present divided into five classes, of which the curable and industrious form the first class, or those who are chiefly employed at needlework, or other sedentary occupation, and amongst them are to be found many of the most useful patients, but some of whom are excitable and at times very troublesome; their total number is about 45. The second class includes chiefly working patients, who are employed in the laundry, kitchens, and out of doors, consisting principally of cases of mania, monomania, and imbeciles, some epileptics, and perhaps a few convalescents; these are upwards of 40 in number. The third class (which with the two former is on the ground floor) consists of the noisy, violent, and those of disagreeable and destructive habits, including maniacs, some epileptics and idiots, averaging about 33 in number. The fourth class includes the chronic and infirm, cases of dementia, melancholia, some epileptics and imbeciles, and are about 50 in number. The fifth class, the sick or infirmary patients, about 20 in number."

Respecting employing and amusing the afflicted inmates of this asylum, we are informed that

"The greater number of the patients have, as usual, been employed. The boundary wall has been nearly completed, and also the paving of the farm-yard, the stones for which have been quarried and dressed in great part by the patients; and two acres of the land in front, heretofore entirely unproductive, have been cleared and added to the farm.

"Excursions have been made by the best conducted and most industrious male and female patients, as an encouragement, under the charge of the attendants, as usual, during the year. The dances have been continued at regular intervals, and on one occasion during the summer *all* the patients, with the exception of the most infirm, were assembled; the room was quite full, 265 persons being present; only one male patient had to be removed, the others conducted themselves with the utmost order and decorum, and seemed pleased with the evening's amusement. Additional musical instruments have been allowed by the visitors to be purchased, and five of the male attendants and two male patients play for the dancers, and contribute very much to the enjoyment of the meetings. It has always been the custom in this asylum to associate both sexes in the amusements and recreations, without which they would be comparatively dull and monotonous. A further addition to the books has been made for those patients who can amuse themselves by reading. It is intended also to render the corridors and day-rooms more cheerful, by placing in them maps and engravings; but a strict attention to economy must for the present render the progress of improvement in this respect slow. Everything should be done to give the house the air of freedom, by removing all painful and depressing associations, and by keeping the minds of the patients occupied, with as few intervals as possible, by easy work and occasional recreation."

We quote *in extenso* Dr. Boyd's valuable remarks upon several points connected with the morbid anatomy and management of mental diseases, and the "medical treatment of insanity," contained in the second part of the document now under review.

"The epileptic female who was discharged relieved, having received benefit from a tincture of sumbul, as mentioned in last report, has had a return of her fits, which she believes would not have been the case if she could have obtained a regular supply of that medicine; her fits have not been so severe as formerly, and it has not been found necessary to send her again as a patient to the asylum. Her daughter, my informant, only 18 years of age, has been admitted this year with acute mania, and is now convalescent, and likely soon to be discharged recovered. Most of our epileptics, male and female, have had a trial of the tincture of sumbul in doses of half a drachm to a drachm twice a day. None of them have been permanently relieved; in some their fits have been less severe, and for a time less frequent. The same medicine has been beneficial in some hysterical cases, and there is no doubt that it is a valuable addition to our antispasmodic drugs, with the advantage of being much less disagreeable to the taste than many others.

"Epilepsy, especially when combined with insanity, is considered incurable, but as we are often unable to discover any organic lesion, we should still hope that this disorder will not always be incurable.

"The recoveries in insanity amount to from 60 to 70 per cent. in those hospitals which exclude chronic cases, and cases with organic disease of the brain (paralysis) and epilepsy: whereas in those asylums which admit all cases of insanity indiscriminately, the recoveries amount only to from 20 to 30 per cent.

"Two of the epileptic females present peculiar appearances; both having hemiplegia, are crippled, one on the right, the other on the left side; the right wrist in one of them is much contracted, the hand almost useless, the arm and thigh on that side each two inches smaller in circumference and shorter than the opposite limbs; and both walk with a limp. These peculiarities in the limbs have existed in both from childhood. The fits are less severe in these cases, and they are sharper in their intellects than the other epileptic patients; one of them has learned her alphabet and to spell since she has been here, and has shown an inclination to learn more. They are both passionate and spiteful, and one of them not so fond of learning is particularly given to thieving. I have not seen any notice of similar cases in any writer on the subject; but I have had several opportunities of examining the bodies of such patients after death, and have invariably found a deficiency in one hemisphere of the brain; and in nearly every instance, in the side opposite to the paralyzed one. The most remarkable case of the kind which I have examined occurred in 1842; a man, aged 47, fell down in the street in a fit, and died shortly afterwards. The left arm was very much smaller than the right, the wrist and elbow contracted; the left thigh and leg were also very much smaller and shorter than the right. It was ascertained that he had always been lame on the left side, with the arm contracted; that he was slovenly in his habits, a miser in disposition, and an inordinate eater; that his business had been that of a linen-draper; that he was considered a good accountant, and had been at one time a lawyer's clerk. On making the post-mortem examination, a large quantity of fluid, which was contained in an anormal membrane over the right hemisphere of the brain, escaped from the same side of the head; the convolutions on that side of the brain were wanting, and the hemisphere was only half the size and absolutely half the weight of that of the opposite side: the right hemisphere of the brain weighed 9¼, while the left hemisphere of the brain weighed 18½ ounces, which last is not above the average weight of that part in the adult male.

"As previously stated, I have found that the average weight of the brains of the insane is above the average weight of the male and female brain of the sane above puberty. In 133 insane adults, 79 males and 54 females, examined in this institution, the average weight of the brain in the males was 47, and in the females 43¾ ounces, being one twenty-fourth part heavier than the average weight of the brain in the sane.

"A reference to the obituary will show that there have again been several cases of general paralysis, accompanied by the usual inflammatory softening and sometimes induration of the spinal cord, and generally of a part of the brain itself. In most cases the disease appeared to have originated in the brain, the mental faculties having been observed to be weakened before the paralytic symptoms manifested themselves: in some instances, the paralytic symptoms were first observed and the mental weakness succeeded.

"The last of the female cases but one, in the obituary, is singular. She had been a cripple for several years, her knees firmly contracted; when sitting up, they were on a level with her chin, and her heels close to the backs of her thighs. The lower portion of the spinal cord, to the extent of 1½ inch, just above the tail continuation, was dark coloured and softened; a portion of it was submitted to microscopical observation by my friend Mr. Gulliver, who, without knowing anything whatever of the case, described it as differing from the inflammatory softening most commonly occurring with the characteristic exudation-corpuscles in the cases of general paralysis. He found that the ultimate nervous structure was merely *disintegrated*, or broken down,—a simple solution of continuity of which Dr. Bennett has described examples in the brain.

"The last female case in the obituary is also of interest, as an example of the protracted period to which a person may live without any great degree of bodily suffering, with extensive ovarian disease, for the removal of which a highly dangerous surgical operation has been of late years too frequently performed. This person died in her 57th year; the disease was reported to have been apparent 39 years before her death. The ovarian cyst was 42 inches in circumference, and contained three and a half gallons of purulent matter. A small cyst was attached to the base of the great one, and contained about one pint of serum."

According to the tenth report of the physician attached to the Dorset County Lunatic Asylum at Forston, the number of resident patients was 164, of whom 68 were males and 96 females; the new admissions during the preceding year having been 51, comprising 23 male and 28 female lunatics. Respecting these inmates—

"Hereditary predisposition was ascertained to exist in six cases; but in all probability this was far below the actual number. The extreme reluctance of friends to admit the existence of insanity in their family, prevails very generally in all ranks of society; and yet probably there are few families in whom this direful calamity does not, immediately or remotely, to a greater or less extent exist.

"A suicidal disposition displayed itself in six of the cases admitted, and one attempt at self-destruction has been made during the year; and one had, previously to admission, inflicted an extensive wound in the throat by means of a knife.

"These cases necessarily cause great anxiety, and considerably increase the duties of both officers and attendants. Providentially the attempt made was defeated; still it is an ascertained fact, that the suicidal propensity sometimes returns suddenly, and quite unexpectedly, after a long interval, and the patient, when not suspected of harbouring any such design, will instantaneously be overpowered with the desire of putting an end to his existence. No vigilance, however incessant and philanthropic, can always successfully guard against the occurrence of such a catastrophe."

As to the cases admitted—

"Several of these have been of a very unfavourable nature. It will be apparent, that even those represented to be of recent date, were, in many instances, in a state of such extreme physical debility as, from the mental disorder being complicated with organic disease of other important viscera, rendered their case all but, if not quite hopeless. It was evident, on their admission, that there was not the least chance of any benefit being derived from medical treatment; in fact, in some instances, stimulants were immediately resorted to as absolutely essential to prevent their sinking from physical exhaustion; and all that could be done for them was to render the brief residue of life as comfortable to them as possible."

In reference to those discharged, it appears that

"Twenty-eight cases have been discharged during the current year. Of the 20 admitted within three months of the invasion of the disorder, 40 per cent. have recovered. Of the remaining, 4 were cases in which the insanity was complicated with paralysis or epilepsy; 2 were the subjects of senile insanity, of whose recovery no rational hope could be entertained. Of those who were admitted for the second or subsequent attack, and within three months of that attack, 42·85 per cent. have been discharged. This is a most gratifying result, when the melancholy fact is borne in mind, that even in those hospitals where none but recent cases are admitted, and of them none but such as are deemed to be curable (the paralytic and epileptic being inadmissible), one half of the patients so admitted are reported or considered to be incurable by any human means.

"It must be remembered, too, as was observed in last year's report, that many young persons, after a violent maniacal attack, fall at once into a state of incurable unsoundness of mind; and that not *a few* others, in consequence of epilepsy, become gradually and hopelessly imbecile. Of these cases, there exists little if any hope of the patients' restoration to reason.

"Where the insanity existed a year previously to admission, one recovered. The importance of sending patients as early as possible to the asylum after the first symptoms of insanity exhibit themselves, cannot be too strongly insisted upon; nor can the pseudo-humane procrastination of those on whom devolves the care of such individuals be too strongly condemned."

Again, regarding the deaths, it is stated, that

"The asylum has, during the year, been entirely free from any epidemic, and the patients have been remarkably healthy.

"7, or 53·84 per cent. of the deaths were occasioned by general paralysis or epilepsy; 2 from disease of heart; and three from decay of nature, aged 86, 88, and 90 respectively; 5 of the deaths occurred from general paralysis of the insane; in 1 it slowly and progressively developed itself, extending over a period of five years from its first development; in the other, it ran a rapid course of a few months. Under the ravages of this formidable and hitherto invariably fatal malady, they all sank into a state of extreme debility and irremediable decay. They were in a state of dementia, and subject to attacks of epilepsy.

"The deaths amount to 8·05 per cent. of the average number resident. This forms a small rate of mortality. General paralysis, epilepsy, and senile exhaustion, have proved the immediate cause of death, in the largest proportion of the fatal cases. Of the 5 patients who died from general paralysis, only 1 was a female."

Several other questions of interest are discussed in Dr. Button's able report; but our limited space only permits quoting his remarks on employment:

"It is a well established fact, that nothing tends so much to the restoration of the mind to its proper tone, as healthful labour and full employment. Continual occupation of the mind on some given subject is one of the best means of drawing off the attention from imaginary or personal sufferings, and of preventing the mind from passing into a state of reverie or abstraction. It also raises the sufferer, especially in the incurable class, above the depressing influence of physical disorder. By the employment of the patients in agriculture, a greater quantity of productive labour is put in motion than can be effected by any other means; and in proportion to the quantity of productive labour called into action, not only is the value of the land increased, but the actual expenditure of the institution diminished. Of all the ways in which patients can be occupied, this is by far the most advantageous. The farm and the garden, the laundry and the kitchen, the shops of the shoemaker, the blacksmith, and the carpenter, continue to be found valuable, as furnishing a means of occupation by which the recovery of the convalescent is advanced, and the incurable relieved from the misery attendant on a state of idleness.

"The beneficial effects resulting from occupation can scarcely be over-rated. Farming and gardening are both useful and pleasing to the insane. They afford the kind of employment which they can profitably follow, both for themselves and for the institution in which they are confined. There are few patients who cannot be employed in some kind of out-door labour; even the imbecile and the demented can be brought to dig and plant, or wheel a barrow; and thus something of interest or pleasure is found to engage them when allowed to be with others who are at work in the field. Active exercise in the open air invigorates the frame, improves the appetite, occupies the mind, and prevents it dwelling on its varied hallucinations. The exercise of the day is followed by sound and refreshing sleep, the patient awakes calm and tranquil, and his mind is composed. Whatever lessens the excitement of the nervous system, and tends to restore composure, must be considered a means of relief, and the means of promoting a more speedy recovery."

At the Devon County Asylum, where the number of lunatics resident on the 1st of last January, amounted to 428, 119 new patients labouring under insanity have been admitted, comprising 63 males and 56 females, during the previous year; whilst 52 had been discharged recovered, and 47 died, of whom 29 were males and 18 females; thus giving a mortality of 39·49 per cent., if calculated according to the admissions. A peculiar feature in reference to the total deaths at this institution deserves special mention—namely, that nearly one-fourth of the mortality arose from general paralysis. Upon this very serious malady, which is now nearly as well understood in England as it has long been by the psychologists in France, Dr. Bucknill says—

"I frequently admit patients with early symptoms of this disease whose mental disturbance is slight, and whose friends have been encouraged by medical men to expect for them a speedy recovery—a hope, vain indeed; for there is no malady in the whole category of human ills more incurable, and more inevitably fatal than the one in question. But a groundless expectation of cure is not the greatest evil which ignorance of this malady may occasion; for its insidious nature may expose those suffering from it to unmerited treatment."

In a subsequent paragraph respecting this malady, the reporter further observes:

"Patients in the advanced stages are liable to an accident of an alarming and dangerous character, the proper treatment of which merits consideration. In consequence of paralysis of the muscles of deglutition, a morsel of food is apt to stick in the pharynx, and impede or stop respiration: these patients have generally excellent appetites, and require substantial diet, the solid part of which should be cut for them into pieces less than an inch square. After this is done, in their endeavour to swallow several pieces together, they are liable to become choked in the manner described.

"When such an occurrence takes place, the attendant has instructions to pass his finger with promptitude and decision into the pharynx, and remove the obstructing mass; not a moment must be lost, even to send for medical aid. Though such accidents have frequently occurred in this asylum, in two instances only did they terminate fatally. Both of these occurred in the spring of last year.* In both surgical aid was immediately rendered, and tracheotomy performed in vain. One patient respired several times through the opening, and then sunk, the shattered nervous system being incapable of reaction. By examining the condition of the parts in these cases, I ascertained that a portion of the food had been drawn into the opening of the glottis and impacted there, accounting thus for the want of success in attempts to remove it. The impeded act of deglutition had taken place while the lungs were empty, and violent inspiratory efforts had drawn a portion of the morsel into the opening of the windpipe. The reflex action of the pharyngeal and laryngeal nerves was impaired, and the morsel consequently was not passed in the right direction by the muscles of deglutition, and was not excluded from the opening of the air-passages by any preservative constriction of the glottis. Should such a case again occur, I apprehend the treatment should be to seize the root of the tongue with a proper instrument, and, drawing it forcibly forward, thus bring the glottis within reach of the finger. It is evident that probangs and the curved forceps would be useless in such cases, and that tracheotomy is likely to cause fatal delay. Restriction to fluid diet would obviate all danger from this source, but could scarcely be used for any length of time without a relaxing and debilitating effect, and a more unfavourable influence on the average duration of life than the occasional, but unfrequent, occurrence of a fatal accident. When an epileptic fit attacks a person who has the mouth filled with food, similar danger occurs; but the attendant has always been able with facility, probably owing to a different condition of the glottis, to remove the food. In one instance, however, I have known an epileptic patient suffocated. He was found dead in bed, with his face upward, the

* These patients were being fed at the time by attendants: the attention of the attendant was arrested by another patient having an epileptic seizure in one case; in the other, by an altercation, and the food being placed down, a paralytic helped herself, and was immediately asphyxiated.

nostrils and mouth filled with a pultaceous mass, formed of bread and curd of milk, upon which he had supped. Vomiting had occurred during the epileptic seizure, and the rejected contents of the stomach formed a complete obstacle to respiration, already impeded by the fit. No food had been drawn into the air-passages."

Another observation made by the same authority also deserves being now quoted, and with which we shall conclude our present notice of Dr. Bucknill's report; not, however, before acknowledging that any similar remarks become especially valuable, when based upon actual experience, as in this instance:

"It has been stated that in general paralysis death occurs always before the end of the third year: This appears incorrect; for, of 50 patients who have died here of this disease, 4 survived to the end of the fourth year; while 2 others are living, after the lapse of the same period. The most rapid cases ran through the several stages in 5 or 6 months; the average duration has been 18 months."

We now approach the public metropolitan institutions for lunatics, including the Surrey Asylum, St. Luke's, and Bethlem Hospital; upon each of which establishments, a few remarks will doubtless prove acceptable to medical readers. Respecting the new asylum at Colney Hatch, we spoke in a recent number; and in reference to Hanwell, we defer any allusion to that institution till a future occasion.

According to the medical report of the Surrey County Asylum, it appears, 359 new cases were admitted during 1851; 116 were discharged cured, and 120 died; hence giving a mortality of 33.42 per cent. if calculated according to the admissions. Of the whole fatal cases 59 were male patients, amongst whom it is interestiug to state, 17 died of general paralysis; whilst out of the 61 females whose deaths were reported 13 also sunk under the same inveterate disease. No male patient died of phthisis, although 4 females became its victims. It is further stated, that 12 males and 14 females were carried off by "gradual exhaustion!" But what this pathological term really means, we are at some loss correctly to understand.

Seeing the medical report of this establishment—where there were under treatment, on the 1st of December last, 853 lunatics, comprising 374 male and 479 female patients—embraces only four pages of large print, the matter from which a critic might make quotations is consequently very limited. Nevertheless, we willingly extract the following observations:

"It gives your medical officers great pleasure to report the recovery of more than ten patients, in whom mental disorder had existed so long, that there appeared but little probability of a favourable termination. In the case of one of the females, the disorder had existed upwards of twenty years. With a little assistance from the Benevolent Fund this poor woman is now able to support herself by making shawls.

"Another female, who had been twelve years insane, on her discharge succeeded to a little property—she now conducts herself with great propriety and respectability.

"A young male patient sent to the asylum, stated to have been imbecile from birth, gradually acquired strength of mind, and learned the trade of shoemaking before he was discharged.

"In the male department, occupation in the farm, garden, and work-shops—and in the female department, needlework of various kinds, and household work, together with instruction and amusements suited to the different sexes, continue to be extensively employed, and to exert great influence in restoring the patients to health of body and of mind, and in rendering the asylum cheerful and comfortable."

Several elaborate tables are appended to the report, but we must here remark, with all deference, that had the large array of facts thus supplied been differently arranged, so as to enable readers easily to draw general deductions, they would have proved much more instructive. It is hoped this hint will be kindly taken, and remedied on future occasions; since the medical officers are fully competent to supply any deficiencies.

At St. Luke's Hospital, 196 curable patients were admitted during 1851—77 being males, and 119 females; whilst 41 male and 90 female patients were cured, and 8 males and 5 females died; thus giving an average per centage, male and female lunatics taken together, of 74 cures amongst every hundred admissions; the ratio of deaths being 7·35 per cent. These results are satisfactory, especially in regard to the large number of recoveries, which thus constitutes a much higher proportion than that usually reported from many other institutions for the insane.

After alluding to the facts now detailed, the physicians next remark,

"While thus recording this unusually large proportion of recoveries, it becomes us to acknowledge that there have been circumstances in the past year which may have given an impulse to such a result, more especially among the female patients. The Centenary Anniversary undoubtedly attracted much attention, and the interest which was manifested in its success among the governors was communicated to the officers of the charity. This probably animated the curiosity and stimulated the energies of the patients. Those who watch the manifold associations which, either for good or for evil, influence the diseased mind, know well how to appreciate such emotions, and to direct them into a proper channel; and it is not too much to say, that some of the recoveries which have been noted in the past year, may with justice be attributed to the knowledge which the patients acquired of the exertions which were being made in their behalf. The chord of hopes long past may have been touched, the germ of sympathies long buried may have been quickened, and if so, how ample is the reward of those who have endeavoured to ameliorate the condition of the inmates of the charity, when they reflect that, by God's blessing upon their labours, more than the usual proportion among the patients have been restored to their happy home."

When speaking of the general management of this hospital, the same authorities likewise observe—

"The balls, the library, and the different occupations and amusements of the patients, fall more immediately under the superintendence of the resident officers; and we therefore take this opportunity of saying how efficiently they have laboured in the various departments allotted to each.

"The weekly average number of patients attending divine service has been greater this year than the last, and it will probably be greater still in subsequent years, as the accommodation in the chapel recently erected is so superior to the old building appropriated for the purpose. Our worthy chaplain devotes himself to this holy work with much zeal and discretion—a task of no small difficulty—for in insanity the higher faculties of the intellect, and the better feelings of the heart, are generally suspended; and it is necessary to warn those who associate with the insane, to take care lest they should imperceptibly be induced to lower their standard of responsibility, and suffer themselves to imitate in any degree the uncontrolled passions of those whose disease renders them irresponsible, while at the same time it is essential to recal the healthy affections of the patient, to soothe his fears, to correct his desires, and to avoid stimulating his imagination, already too active."

Numerous statistical tables are appended to the report drawn up by the physicians; and we would direct particular attention to that containing an account of the *post-mortem* appearances met with in those cases who died in the hospital.

The elaborate statement of the weights of patients discharged is also interesting, besides several of the other returns; hence it appears both Dr. Sutherland and Dr. Philp, the physicians, as also Mr. Arlidge and Mr. Walker, who drew up the analyses and statistical tables, deserve credit for the accuracy of those documents.

According to the annual report of Dr. Monro and Sir A. Morison, Physicians of Bethlem Hospital,

"The number of curable patients admitted during the year was 286, consisting of 112 males, and 174 females.

"There is a considerable diminution of admissions during the year, 1851, which is well accounted for, by the consideration that numerous county asylums have been erected, and are in progress, in many parts of the kingdom, which will necessarily diminish, in a material degree, the applications for admission into this and all similar institutions."

Respecting the amount of recoveries, it appears

"The number of patients of this class discharged cured during the year has been 121, consisting of 52 males, and 69 females; being below the usual average—a point which has been considerably influenced by the reduced number of the cases under treatment."

Again, in reference to mortality, the same authorities state:

"The number of curable patients who have died has been 26, viz., 9 males, and 17 females: and as compared with the preceding year they are 5 fewer. If compared with the number of cases under treatment, the proportion will not vary materially. One great cause of mortality will be found to arise from the charitable admission of aged and weak patients, who, perhaps, are not strictly admissible at all, but the governors are loth to reject, when any, even the least hope of a beneficial result may be anticipated."

General treatment next occupies attention; upon which subject the reporters say,

"Many improvements have taken place with regard to the cheerfulness of the wards, especially by enlarging the windows in the basement, which gives a much more agreeable air than heretofore to this part of the hospital. The fire-places have also been improved, and as far as the comfort and appearance go, are much more appropriate. But great care will be necessary to prevent accidents, especially on the female side, as the frequent negligence of sane persons with reference to this risk, is of course materially increased in those who are labouring under mental incapacity."

"Several side rooms, of large dimensions, have been added to the previous accommodation of the patients, which are of a very airy and spacious character, and constitute a very agreeable addition to their comfort.

"The use of knives and forks has been continued without any accidents.

"A new airing-ground for the use of the basement patients on the male side is very serviceable, as detaching the worst cases from the more orderly, and thereby insuring the comfort of such as are well disposed. The new ground is not very large, and is too much exposed to the gaze of neighbouring windows, but still it is a valuable addition."

As to amusements, and the use of restraint at this establishment, we are informed that—

"Much time is devoted by a large proportion of patients to various occupations, and the needle rooms on the female side have been multiplied.

"Now and then a piano enlivens the scene. The men are, many of them, engaged in the gardens and galleries, and a few in the shops. The average number of patients in employment has been $243\frac{215}{311}$.

"The amount of seclusion has been very small, amounting only to $\frac{126}{365}$. Occasional seclusion of clamorous or violent patients has been adopted, as preferable to any other mode, and it has been very beneficial in some instances. In point of fact, there has been *no* mechanical restraint exercised throughout the year, but simply occasional retirement of unruly cases."

This statement is gratifying, as evidence of a disposition to introduce ameliorations in the management of an institution at present unfortunately under a cloud, in consequence of the late investigation by the Commissioners in Lunacy, but respecting which it is anticipated a satisfactory explanation will be speedily forthcoming.

Numerous statistical tables of the same description as those appended to former medical reports, are subjoined to the present document. All are very elaborate and valuable. Indeed, few seem more so, whether the facts thus registered are examined separately, or the important deductions which may be obtained, when viewed in the aggregate, are taken into consideration; but especially, if a series of years be embraced in such calculations. Without undervaluing medical observations based on practical experience, we would still assert, that accurate and well-arranged statistical tables—derived from large institutions—constitute an excellent "pabulum" for the mental digestion of all lovers of exact knowledge.

Until within a few years, Wales was almost wholly devoid of public institutions for the insane. Very recently, the North Wales Asylum was established at Denbigh, of which the third annual report by the medical officers now lies on our table. According to this official document we find that—

"During the year 1851, the admission of patients amounted to 72; the recoveries 19; the improved 5; the deaths 11.

"When it is considered that only three years and a few months have elapsed since the institution was opened for the reception of patients, and that nearly all the hopelessly insane belonging to the five counties in union, who had been for years confined in other asylums, constituted a large proportion of our inmates, the majority of whom still survive; and that several of those more recently admitted, were chronic and neglected cases—putting aside these poor creatures, and calculating our recoveries upon the data of recent admissions, our tables will show as large a proportion of cures as most institutions of a similar kind in the country."

Being a new establishment, considerable improvements remain to be accomplished, of which, Dr. Williams the visiting physician, and Mr. Jones the superintendent, make prominent mention; as, for instance, when they emphatically remark that

"Since the commencement of the present winter, we have become more and more convinced of the injury the institution is suffering from the want of a proper mode of lighting the establishment. We have visited many institutions of a similar kind since our last report, and it has been with much more mortification and regret that we have contrasted the brilliancy and comfort which pervades other asylums, at night, with the gloom of that, over which we preside. We feel assured that a visit to our house between the hours of half-past four and nine o'clock, in the long winter evening would convince any one having the least regard for its prosperity, of the truth of our statement. Our long galleries and passages are nearly dark, and the sitting-rooms only enjoy the glimmer of an oil lamp, by no means adequate to give sufficient light for occupation of any kind. Were we supplied with gas, our poor patients could usefully employ themselves in reading and other recreations during the dreary hours which intervene between dusk and bed-time; and what a happy change would its bright and cheerful light afford to the "moody melancholic," after the gloom of a wet and dreary winter's day! The approach of evening would then be hailed as the most agreeable and cheerful part of the day—as it now is the most dreary. The increasing number of private patients cannot be expected to be progressive, unless we afford them the means of agreeable occupation during the long winter evenings.

"If we have expressed ourselves warmly upon the point, we trust we may be held excused, as we are pleading the cause of those who cannot plead for themselves, and for whose comfort and prospects of recovery we are in great measure responsible. It is discouraging to us to find that all our anxious efforts to render the North Wales Asylum a model in the Principality, should be thwarted for the want of the ordinary appliances and remedial means possessed by almost every other asylum in the kingdom.

"Another very leading defect in our institution is the want of workshops, placed and secured from the chances of escape. We are now obliged to set apart one of the day-rooms on the men's side of the house for a working-room for tailors and shoemakers. The rapidly increasing number of our patients will very soon oblige us to occupy this apartment as a day-room or a dormitory. One of our galleries is now used as a carpenter's shop, which being a thoroughfare, renders it open to much objection and danger. There are several patients who might be usefully and profitably employed as carpenters, joiners, &c., had we the means of occupying them in properly secured rooms. The situation and cold of the mangling-room continues a source of great complaint."

Amongst the 72 new patients admitted during the past year, 23 laboured under acute mania; 31 had chronic mania, 9 dementia, 3 melancholia, 2 epilepsy, 1 general paralysis—a male, and 3 were suicidal. Respecting the 11 deaths reported, it is interesting to learn that 8 died of chronic mania, 2 epilepsy, and 1 by general paralysis; whilst the number of lunatics remaining under treatment on the 31st of December, 1851, was 156, both sexes included.

Scotland has long maintained a high position in reference to its public institutions for the insane, as well with regard to the proper treatment of lunatics, as the general management of those establishments. The first asylum we shall notice, in the present analysis, is that of Dundee, of which the thirty-second annual report, signed by Drs. Nimmo and Wingett, was presented to the directors on the 21st of last June. In this document these physicians state :

"The principal results of the year are comprised in the following summary :—

"The number discharged recovered has been 60 per cent. of the cases admitted.

"The number of deaths has been 4.85 per cent. of the daily average number of patients resident.

"No serious accident has occurred. And it is worthy of mention, as an indication either of carefulness on the one hand, or of composure upon the other, that no one has passed beyond the grounds of the asylum without permission for so doing.

"The above ratios entitle the past year to be congratulated as one of prosperity. A retrospective glance at the records of the institution justifies this. The total number discharged cured during the whole career of the asylum, is 45.39 per cent. of the total admissions. The obituary for the last twenty-two years shows an annual mortality of 5.93 per cent. of the daily average number resident."

In another page of their report the same authorities likewise remark—

"Forty patients have been admitted during the year. Admission has been refused to 14, in consequence of want of room to accommodate them. The duration of the disease previously to admission varied, in those admitted, from one week to twenty years.

"Twenty-four individuals have recovered, and left the institution during the year. The duration of their residence ranged from three months to twenty-seven years. The patient who passed so many as twenty-seven years as a resident here, had been a coach-driver by occupation, and was affected with intermittent mania. It may seem strange to one unacquainted with the social characteristics of the institution, to know that this man took his departure with much reluctance. He said—'I have, however, one consolation, if I grow ill again they will send me back to you.' He was 68 years of age when he left the asylum. Recovery, after so many years of mental affliction, is not of frequent occurrence. It is, however, most cheering to know that it may and does occur, and that the term incurable as applied to insanity, must be received *cum grano salis*.

"Ten patients have died during the year, viz., 5 males and 5 females. Among the causes of death no acute inflammatory disease has occurred. Of the five male patients who have died, one was in so deplorable a state of exhaustion from inanition, maniacal excitement, and wounds produced by the measures resorted to by his friends for confining him to his bed, that his life was despaired of at the time of admission, and he died one month afterwards, aged 40. The second, aged 72, died of apoplexy, after having resided thirty years in the asylum. He had passed those years in a state of fatuity, little conscious of passing events, and subject to occasional paroxysms of excitement. The third, aged 37, died of heart disease, after fifteen years' residence. The fourth, aged 25, during a fit of epilepsy; and the fifth, aged 46, of general paralysis. Of the five females who have died, the first, aged 59, was from exhaustion, following protracted excitement, her constitution having been previously much debilitated by purpura and inanition. The duration of her residence was four months. The second, aged 55, of marasmus, following severe chorea, after five years' residence. The third, aged 77, of heart disease, after fifteen years' residence. The fourth, aged 30, during a fit of epilepsy, after seven years' residence; and the fifth, aged 58, of exhaustion, induced by intense suffering from melancholia of two years' duration."

Some judicious observations are also interspersed in other portions of this official statement, but we must confine our quotations to the following concluding paragraph:

"In watching the progress and results of treatment in this institution, there is one circumstance which, although apparently of insignificant importance, has really a great deal of influence in augmenting the general prosperity. This arises out of the fact, that many classes of patients are here organized into one community; that although the distinctions of rank are preserved, separation as regards residence effected, and the privileges, enjoyments, and pursuits of all have their appropriate limits assigned to them, there is, nevertheless, a certain amount of voluntary communication among the different grades highly conducive to the health and happiness of all. For instance, in our little community there is a great deal of patronage exercised by the rich to the advantage of the poor. Rank and fortune have their protegées; and at the same time, that the poor are benefited and encouraged by these kindnesses, the donors find both occupation and gratification in the act. Many of the poorer classes of patients are capable of becoming very amiable and amusing companions, and regard an invitation to the parlour of a superior in rank as a flattering compliment, and such visits generally end in the gift of some article of wearing apparel, or some confectionery, or other luxury, or the loan of books and periodicals, but always in a reciprocity of benefits by the exercise of courtesy on the one hand, and gratitude and respect upon the other. Another advantage resulting from this incorporation of classes consists in the opportunities which are thus afforded to the upper classes of co-operating with, or learning from, the humbler residenters, any manual occupation to which their own inclination leads them, or which may be prescribed to them as a remedial measure. These mutual benefits flow naturally from the constitution of the asylum, and afford so many auxiliaries for promoting harmony and happiness."

The Glasgow Royal Asylum next presents itself to our notice. There the total number of lunatics remaining on the 31st of December, 1850, was 425, of which 227 were males, and 198 females; thus giving a predominance of mental disease amongst the former over the latter sex. According to the physician superintendent, Dr. Mackintosh, formerly of the Dundee asylum, and where he had already earned a high reputation—

"The new cases in 1851 fell much short of those in 1850. In 1850 we had 393 new cases, and only 259 in 1851; being a decrease of no less than 134. This decrease is confined to the lowest class of patients. The number of private patients, especially those at the higher rates of board, have been gradually increasing; so that, while at the end of the year 1850 the number of patients at the higher rates was 85, notwithstanding the increase of deaths and dismissal in the West House, at the end of the year 1851 it had increased to 102."

Amongst the male patients admitted the prevailing forms of insanity were mania and dementia; whereas, in the females, melancholia predominated. Again, respecting the assigned causes of their mental malady, intemperance stands prominently forward; 54 cases out of the 259 admitted, having been occasioned by that sad propensity to whiskey drinking, which prevails to such a lamentable extent throughout the lower orders of the population. Disappointed love produced madness in 12 inmates, 9 being females, but only 3 males. Puerperal causes were reported in 10 females. Religious excitement in 9 individuals, whilst 43 cases—16 males and 27 females—were ascertained to have laboured under previous attacks of insanity.

Respecting the proportion of recoveries, the report says:

"The table of cures shows a total of 110 during the year. The disease in no less than 90 of these cases had been of shorter duration before admission than one month—a circumstance strikingly illustrative of the great necessity of placing the patient under medical treatment at its very first appearance, or at least very shortly afterwards. Even in a pecuniary point of view, whether as regards relations or parochial boards, how great is the difference between an early and a late appliance of medical measures! If taken at its commencement the disease, in perhaps four cases out of five, will probably terminate favourably in the course of a month or two; but if it has been neglected for any length of time, the chances of convalescence are reduced so low, that, in a few instances, years may elapse before recovery takes place; while, in the great majority, the patient lingers on in a state of utter mental imbecility till death closes the scene."

The fatal cases during the year amounted to 42, of whom 14 died within two months after the patient's admission, and in reference to the apparent causes of death it is stated, that,

"One patient died from disease of the spine. She had been eight years an inmate of the house. About February last she gradually lost power over the lower half of the body; soon afterwards, a curvature of the upper part of the dorsal portion of the spine appeared, and latterly increased considerably. She lingered for about ten months in a state of great helplessness, her sufferings being much aggravated by torpor of the intestines.

"The deaths in the East House amounted to 30; in the West House they amounted to 12. Of this number, 9 were from general paralysis; 3 in the East and 6 in the West, or 1 in 10 in the East and 1 in 2 in the West House, or as 5 to 1 in the West House compared with the East. This remarkable difference leads to the inference, that this form of disease is more prevalent among the wealthier and better-educated classes of the community, and may possibly be explained by the dissipation, in some, of the mental energies in frivolous pursuits; or by their over-exertion, in others, in the active business of life."

Throughout the period embraced by this report, the patients in general enjoyed good bodily health; and, further, it is stated, that,

"During the whole year, no case of contagious or epidemic disease occurred. In the first quarter of the year there were a number of cases of influenza, and attacks of inflammation in the respiratory organs. In autumn there were, as usual at that season of the year, many cases of bowel complaint, some of which terminated fatally. The very severe weather at the end of the year produced, as usual, a number of cases of catarrh and bowel complaint, though not more than what are usually met with at that season. A few whose constitution had been much weakened by previous suffering, sank under them."

When speaking of the treatment adopted, Dr. Mackintosh observes,

"In order to maintain the general health of the patients as much as possible, I was obliged, during the very hot weather in June and July, to restrict considerably the usual out-of-door exercise between the hours of 9 a.m. and 6 p.m. The want of suitable sheds in the airing courts was the principal cause of this restriction. I hope that during the course of the year 1852, it may be in the power of the directors to erect a number of them, as they are of very great importance for the safety and comfort of the patients. Every care was taken to prevent those who were allowed to go out, from sleeping on the ground or exposing their heads to the powerful rays of the sun; and I am happy to say, that, from the precautions taken, none of them suffered in any way.

"In May, Dr. Fleming performed the operation for strangulated femoral hernia, on a female of advanced age, with his usual ability and success.

"In regard to the medical treatment of the different forms of insanity, the usual remedies were applied, varied, of course, in every case according to its peculiar circumstances. The usual moral and physical means of treatment, combining amusement, exercise, and instruction, were all in constant operation whenever a patient was in such a state as to be benefited by any of them. The magic lantern was for a time a great fund of amusement. During part of the year the patients had weekly concerts of vocal and instrumental music. All strangers were strictly excluded from these meetings, which were invariably confined either to ladies alone, or to gentlemen, so far as the West House was concerned, so that no offence of any kind might be taken by the relatives or guardians of the patients. Much of the credit of these meetings is justly due, and cheerfully given, to a gentleman whose name I am not at liberty to mention, but who, after an illness of nearly four years' duration, has so far recovered that he will likely in a few weeks be dismissed as cured."

Various medical statistical tables are appended to the report from which the above extracts have been made. These official statements, like others already mentioned, when speaking of several public institutions previously passed under review, contain many interesting facts which deserve perusal by physicians occupied in similar inquiries.

Amongst the public institutions for lunatics in Scotland, the Royal Edinburgh Asylum, whilst one of the most recently constructed, is also, we believe, the largest establishment of the kind belonging to that country. By the last annual report,

"The number of patients admitted during the past year was 248. At the close of the year 1850, there were 498 inmates, so that there have been 746 patients under treatment, since the date of the last annual report. Of these, 218 have been removed by death and otherwise, leaving at the close of the last year 516 patients in the house. The average number resident during the year was 520, being an average of 26 more than the previous year.

"Of the patients removed, 111 were cured, being in the ratio of 44·8 per cent. to the number of admissions, or of 21·3 per cent. to the mean number resident.

"The number of patients admitted into the asylum since its foundation is 2670, and the number removed cured is 1100, being 41.2 per cent. of the whole, or 51·3 per cent. deducting those who still remain under treatment."

Speaking generally, the greatest proportion of new patients received were afflicted with mania, of which there were 67 cases out of the 248 admitted; 48 laboured under dementia; 39 exhibited different forms of monomania; 30 melancholia; 13 epilepsy; and 10 were examples of delirium tremens. The cases of general paralysis being fewer than in former years, as also those of puerperal mania.

Respecting the varied forms of mental disease affecting patients recently admitted into this asylum, Dr. Skae, the resident physician, says:

"Many of the cases presented features of interest, but of a kind rather psychological than practical. I shall not therefore dwell upon their details here, further than to state that, as has been before remarked, the current topics of the day gave colouring and form to the delusions of the disordered fancy. We have thus had no less than five individuals, admitted during the year, who believe themselves to be the victims of mesmeric agency—an agency certainly not less afflicting than the Satanic possessions and witchcrafts of former times—with which indeed some of the sufferers think it is identical. One sufferer believes himself to be in mesmeric relation of such an intimate kind with a whole family, that he experiences all the sensations of each member of it, eats and drinks (in imagination) when they do, and is sensible to the taste of what they each swallow at every meal. He is compelled to be an unwilling partaker of their very vices, and laments with horror that even during the innocence of retirement, or of sleep, he is pursuing unhallowed courses with parties at a distance, and feels all the bodily sensations of which they are the subjects.

"Three of the inmates talked much of California, and of the bags full of gold which they had obtained from the diggings; and one of them arrived at the persuasion that his body was transmuted into gold. This insane optimism is commonly associated with the most unfavourable and hopeless forms of madness. Its victims, while they are rejoicing over unbounded wealth, and boasting of superhuman powers, are generally sinking rapidly into the most entire paralysis of every mental and voluntary function. Although the numbers of this class have been fewer than for the two previous years, we have now had added to our list of dignitaries two lords, two dukes, one Prince Albert, one angel, twelve divine personages, and one who claims to be greater than a divinity, calls himself 'Thunderbolt,' and who under the persuasion of his supernatural powers, previous to his admission, constructed a boat made entirely of wood, without a single bolt of iron, in which frail bark he launched himself into the Firth of Forth during a storm, and suffered shipwreck on the coast of Fife."

Upon so interesting a subject as the assigned causes of insanity, the same authority further reports that

"Intemperance figures as usual as the most frequent; the disease being ascribed to it (deducting the cases in which the cause was unknown) in 24 per cent. of the admission, or, deducting the females, in 30.4 per cent. of the male patients. Next in order in point of frequency comes mental excitement. Most of these cases were attributed to what is very erroneously called religious excitement, inasmuch as the excitement is generally caused by the absence of religion, and by the superstitious fears of an ignorant and ill-regulated mind. One of the cases included under this head, was that of a young lady whose mind gave way under the excitement caused by preparations for her own marriage to the object of her affections; in another, the excitement arose from the acquisition of a sum of money, and in a third from over-exertion to realize money.

"Reverse of fortune, and domestic griefs come next in the order of frequency. Of these cases one was caused by the dissipation of a wife, two by the death of husbands, and others by the loss of near relatives. In almost all such cases there exist other causes, acting more or less remotely, predisposing to, or as the immediately exciting causes of the disease. Sudden loss of fortune very often is accompanied by recourse to artificial stimulants, and domestic quarrels and unhappiness follow in the train of evils."

In reference to the deaths recorded, Dr. Skae also observes:

"The mortality during the past year has been less than during the four preceding years, and relative to the number of inmates, very much less. In the years 47, 48, 49, and 50, the deaths were 68, 68, 79, and 64 respectively; during the past year they amounted only to 50, although the mean number resident exceeded those of the previous year by 14, and of the three preceding years by 86, 29, and 47. During the four preceding years the deaths were successively 15, 14, 16, and 12 per cent. to the average number resident; during the past year they have been only 9·8 per cent., or 6·7 per cent. to the whole number under treatment.

"Of these 50 deaths, 31 died of insanity, or the diseases immediately causing it, or the exhaustion consequent upon it. Eight died of phthisis, and 3 of dysentery, the most frequent complications of insanity. The diminished mortality of last year is doubtless due in a great measure to a more healthy season, and in particular to the great decrease in the number of cases of chronic diarrhœa and dysentery, as compared with the years preceding it. The number of deaths from general paralysis (an incurable complication of insanity) was 15, being the same as in 1850, when they materially exceeded those of any former year."

The pathological appearances met with in 33 post mortem examinations are next detailed in a classified form, which will repay perusal. Subsequently, the occupation of patients is discussed; upon which important topic the report states:

"Of these the most beneficial, as a means of cure, is undoubtedly active occupation in the open air. The garden, farm, and pleasure grounds have afforded ample opportunities of this kind to the male portion of the inmates of the Western House during the past year. In addition to the ordinary cropping and gardening operations, extensive undertakings in the way of levelling, trenching, extending the lawn in front, forming new roads, laying down new fences, transplanting trees, and other works have been in constant progress; and have afforded occupation to all the able-bodied, from the most intelligent and docile, down to the most imbecile or unmanageable, capable only of wheeling stones in a barrow. The average number employed under the gardener in this way was, during the summer months, above 100. The females have also frequently afforded bands of cheerful and active workers at weeding, picking, and various other suitable out-door occupations. For them, however, the washing-house, and green, and laundry, and the cleaning operations in constant progress, constitute the chief sources of active occupation."

Amusements and intellectual recreations are not overlooked amongst the varied means zealously employed to ameliorate the afflicted condition of the numerous inmates resident at this asylum; since it appears—

"The out-door amusements and relaxations have been continued as in former years, with little variety, but equal frequency and zeal. Walks and drives in the country, pic-nic parties to the neighbouring hills and glens, bathing parties, fishing excursions, visits to reviews, cricket matches, and other sights, followed each other in close succession. Bowls, quoits, skittles, and cricket were the favourite games at home, and of these cricket appeared to effect the greatest amount of good, from the cheerful emulation and active muscular exertion which it calls into play.

"Musical parties in the eastern department, and the weekly concert and ball in the western, were kept up with their wonted spirit and beneficial results. The latter was occasionally varied, as on former years, by extraordinary exertions on the part of the inmates to mark a holiday, or do honour to some distinguished guest. Halloween and New Year's-day had their usual festivities. On one of the weekly assemblies, the amiable and accomplished physician of Hanwell was received by the patients with the respect and courtesy due to his well-known benevolence. On another occasion, Miss Glyn kindly favoured the assemblage with readings from Anthony and Cleopatra; and during the greater part of the year, through the gratuitous assistance of several of the most efficient professional performers from the city, the music was of more than ordinary excellence, and added very much to the interest and enjoyment of the evenings.

"The library of the asylum has continued slowly to increase, partly through the gifts of friends, and partly from the profits derived from our monthly periodical, which still enjoys a remunerating circulation. The newspapers and periodicals have been increased in number, and all these sources of occupation are eagerly sought after and enjoyed."

Statements like these are highly gratifying. But we must, for the present, take leave of Scottish institutions for the insane, as space will not permit our analysis being now enlarged any further, although several other public asylums north of the Tweed deserve mention: such as the Aberdeen, Montrose, and Perth lunatic establishments; besides the Crichton Royal Institution at Dumfries, already so well known to the profession, through Dr. Browne's admirable annual reports, often noticed in this journal.

---

